# Harnessing *Stevia rebaudiana* for Zinc Oxide Nanoparticle Green Synthesis: A Sustainable Solution to Combat Multidrug-Resistant Bacterial Pathogens

**DOI:** 10.3390/nano15050369

**Published:** 2025-02-27

**Authors:** Mohamed Tharwat Elabbasy, Rasha M. El Bayomi, Esraa A. Abdelkarim, Abd El-Salam E. Hafez, Mohamed S. Othman, Mohamed E. Ghoniem, Mai A. Samak, Muteb H. Alshammari, Fahad Awwadh Almarshadi, Tamer Elsamahy, Mohamed A. Hussein

**Affiliations:** 1 Department of Pathology, College of Medicine, University of Ha’il, Ha’il 55476, Saudi Arabia; tharwat330@gmail.com (M.T.E.); dr.maiamin@yahoo.com (M.A.S.); 2 Food Control Department, Faculty of Veterinary Medicine, Zagazig University, Zagazig 44511, Egypt; rmazab@vet.zu.edu.eg (R.M.E.B.); elged2010@yahoo.com (M.A.H.); 3 Department of Biochemistry, College of Medicine, University of Ha’il, Ha’il 55476, Saudi Arabia; mo.abdelkarim@uoh.edu.sa; 4 Department of Internal Medicine, College of Medicine, University of Ha’il, Ha’il 2240, Saudi Arabia; teto2006.km@gmail.com; 5 Department of Health Informatics, College of Public Health and Health Informatics, University of Ha’il, Ha’il 55476, Saudi Arabia; muteb.h.alshammari@gmail.com; 6 Department of Public Health, College of Public Health and Health Informatics, University of Ha’il, Ha’il 55476, Saudi Arabia; fa.alotaibi@uoh.edu.sa; 7 Independent Researcher, Zhenjiang 212013, China

**Keywords:** *Staphylococcus aureus*, zinc oxide nanoparticles, green synthesis, antibacterial activity, antimicrobial resistance, food safety

## Abstract

The rise of multidrug-resistant (MDR) bacteria in food products poses a significant threat to public health, necessitating innovative and sustainable antimicrobial solutions. This study investigates the green synthesis of zinc oxide nanoparticles (ZnO-NPs) using *Stevia rebaudiana* extracts to evaluate their antibacterial and antibiofilm activities against MDR *Staphylococcus aureus* strains isolated from sold fish samples. The obtained results show that the contamination with *S. aureus* reached 54.2% in the tested fish samples (*n* = 120), underscoring the urgent need for effective interventions. ZnO-NPs were successfully synthesized and characterized using UV-visible spectroscopy, FT-IR, XRD, and TEM, confirming their formation with an average size of 15.7 nm and reflecting their suitability for antimicrobial and biological applications. ZnO-NPs exhibited potent antibacterial activity, with a maximum inhibition zone of 24.4 ± 0.4 mm at 20 μg/disk, MIC values of 6.25–25 μg/mL, and MBC values of 12.5–50 μg/mL. Additionally, biofilm formation was inhibited by up to 92.1% at 250 μg/mL. Our mechanistic study confirmed that ZnO-NPs damage bacterial membranes and DNA, leading to the intracellular leakage of cell components that lead to bacterial cell lysis. The use of *S. rebaudiana* in ZnO-NP synthesis aligns with green chemistry principles, offering an eco-friendly alternative to conventional antibiotics and enhancing the bioactivity of ZnO-NPs, and may address the growing issue of antimicrobial resistance, thereby contributing to improved food safety and public health protection.

## 1. Introduction

Marketed fish are vital sources of nutrition, providing an array of essential nutrients, including high-quality proteins, minerals, and vitamins, which are crucial for various physiological processes [1]. Despite their nutritional benefits, fish products are vulnerable to bacterial contamination, posing significant risks to product quality and public health. Bacterial pathogens often affect species such as Nile tilapia, European sardine, and Atlantic mackerel, leading to significant economic losses from increased mortality and product rejection due to stringent safety regulations [2]. Furthermore, the consumption of contaminated fish can result in serious foodborne illnesses, underscoring the critical need for robust biosecurity measures within the aquaculture industry to safeguard fish health and consumer safety. Among the most concerning foodborne pathogens is *Staphylococcus aureus*, a zoonotic bacterium that ranks as one of the main causes of global foodborne illnesses [3]. The rise of MDR *S. aureus* strains has significantly exacerbated the complexity of treating infections in various fish species, with profound economic and public health repercussions [4,5]. These resistant strains are particularly challenging because they can withstand multiple antibiotics, rendering conventional treatments ineffective. Resistance mechanisms in *S. aureus* are often driven by genetic mutations, horizontal gene transfer, and the misuse of antibiotics, further intensifying the public health crisis.

The incidence of *S. aureus* in fish markets has been extensively studied, revealing significant variability in contamination levels based on the geographic location, type of fish, and handling practice. For example, a study in Egypt found that *S. aureus* contamination in marine fish samples was 64% and 67% in *Saurus* spp. and *Pagrus* spp. [6]. This indicates that the handling and processing stages are critical in influencing contamination levels. Another study in India reported a higher incidence of methicillin-resistant *S. aureus* (MRSA), increasing from 29% to 47% from 2009 to 2014, suggesting that poor sanitary conditions significantly contribute to microbial contamination [7]. These studies highlight the crucial role of hygiene and regulatory measures in mitigating the risks associated with *S. aureus* in fishery products, ensuring consumer safety.

Nanoparticles (NPs) have garnered considerable global attention due to their unique and highly desirable properties. Their small size leads to an exceptionally high surface area-to-volume ratio. This characteristic, along with their tunable shapes, compositions, and physicochemical properties, makes NPs highly suitable for a wide range of applications. These applications span diverse sectors, including but not limited to targeted drug delivery, enhanced imaging and diagnostics, innovative food preservation techniques, high-performance electronics, and advanced water treatment methods [8,9]. Among various NPs, zinc oxide nanoparticles (ZnO-NPs) have recently gained attention for their outstanding physicochemical and biological properties [10]. The application of ZnO-NPs in fish processing and preservation presents a promising strategy to enhance food safety and extend shelf life. ZnO-NPs can be incorporated into fish packaging materials or applied as surface coatings to inhibit the growth of bacterial pathogens, including MDR strains of *S. aureus* [11]. ZnO-NPs are generally recognized as safe (GRAS) by regulatory agencies such as the U.S. Food and Drug Administration (FDA) when used within specified limits, making them suitable for food-related applications [11,12,13]. In fish markets, ZnO-NPs could be applied during the handling, storage, or transportation stages to reduce bacterial contamination and improve product quality [14]. For instance, ZnO-NP-infused packaging films or coatings could be used to wrap fish products, providing a controlled release of NPs that target bacterial pathogens without compromising the sensory or nutritional properties of the fish [15]. The safety of food products is indeed a paramount concern. To prevent ZnO-NPs from entering the human body, their application in non-migratory forms can be proposed, such as incorporating them into food packaging materials or using them as surface coatings on fish products [14,16,17]. For instance, ZnO-NPs can be embedded in biodegradable film or coatings that act as barriers to bacterial contamination without direct contact with the fish flesh. This approach ensures that the nanoparticles remain on the surface of the packaging or coating, preventing their migration into the food itself. Studies have demonstrated the effectiveness of such strategies in reducing bacterial contamination while maintaining food safety [14,18,19].

Traditional methods for nanoparticle synthesis, such as sol–gel processes, chemical vapor deposition, and mechanochemical techniques, often come with drawbacks. These include lengthy processing times, high production costs, and significant environmental impacts due to the use of harsh chemicals and energy-intensive procedures [20,21]. The green synthesis of ZnO-NPs using renewable organic extracts such as those from *Stevia rebaudiana* represents an environmentally friendly alternative that avoids harmful chemicals. *S. rebaudiana* is known for its sweetness and variety of biological activities due to its reducing sugars and phenolic compounds [22]. The bioactive properties and rapid high-yield production make Stevia an ideal agent for the biosynthesis of NPs. Moreover, green-synthesized ZnO-NPs have demonstrated effective antibacterial and antibiofilm properties against various pathogens including *S. aureus.* Despite the promising antibacterial properties of biosynthesized ZnO-NPs against various pathogens, there remains a notable gap in research addressing their effectiveness against the MDR strains of *S. aureus* isolated from marketed fish [23]. Several reports have explored the antibacterial potential of ZnO-NPs synthesized through conventional methods or other biological sources [20,24,25,26]. However, few studies have focused on green-synthesized ZnO-NP applications as a targeted approach to combating MDR strains in retailed fish and their products.

This gap is critical as conventional antibiotic treatments continue to lose efficacy against resistant strains. Thus, innovative techniques for nanoparticle biosynthesis and understanding their antimicrobial mechanism can serve as an alternative antimicrobial strategy to enhance food safety. Consequently, this study aims to investigate the use of *S. rebaudiana* as a reducing and capping agent for biosynthesized ZnO-NPs and explore their bioactivity as antibacterial and antibiofilm agents towards virulent MDR *S. aureus* strains isolated from marketed fish. Additionally, this study aims to explore and elucidate the antibacterial mechanisms of these biosynthesized ZnO-NPs, contributing to novel strategies for enhancing food safety in fish markets.

## 2. Materials and Methods

### 2.1. Sample Preparation and S. aureus Isolation

**Figure 1:**
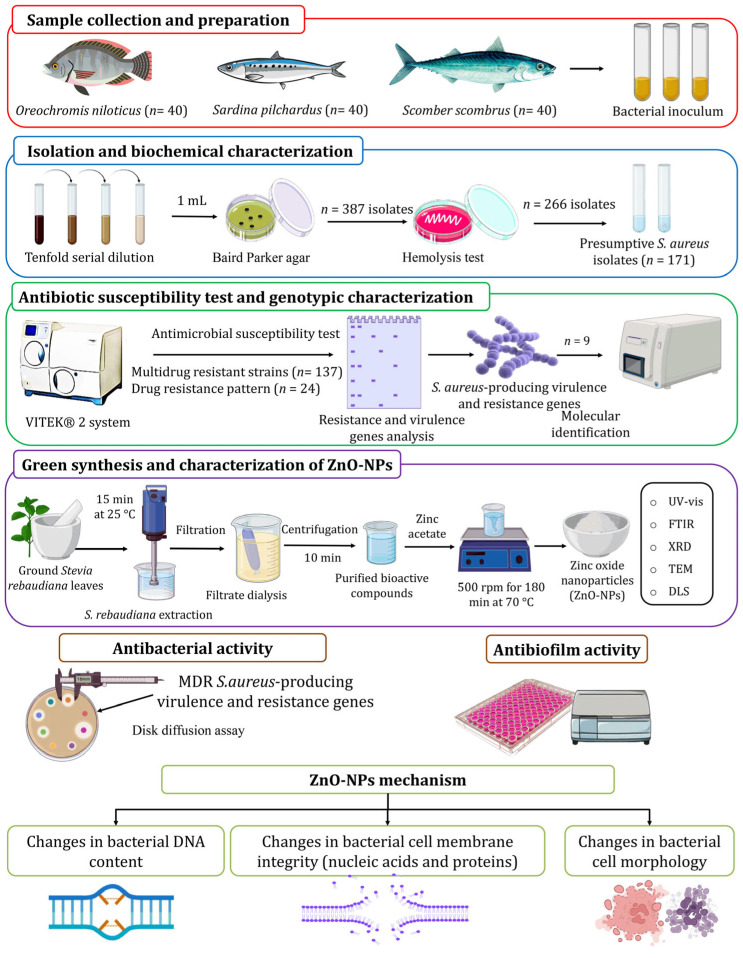
Experimental setup to isolate and characterize pathogenic MDR *S. aureus* from marketed fish samples and explore the efficiency of ZnO-NPs and their mechanism as an antibacterial agent.

Fish samples (*n* = 120) were collected randomly from fish markets in Kafr Elsheikh, Egypt, comprising 40 fish samples each of European sardine (*Sardina pilchardus*), Nile tilapia (*Oreochromis niloticus*), and Atlantic mackerel (*Scomber scombrus*). The fish were transported to the laboratory in aseptic polyethylene bags and subsequently stored at 4 °C. The organs of the fish, including the skin, gills, and gut, were excised and placed in sterile containers for the screening of *S. aureus* contamination. Sterile cotton swabs were used to collect samples from these organs, which were then inoculated into 10 mL of peptone water and incubated for 12 h. As shown in Figure 1, a tenfold serial dilution (10^4^ to 10^8^) was prepared. One milliliter from each prepared dilution was spread onto Baird Parker agar (BPA) enriched with egg yolk tellurite emulsion (HiMedia, Thane, India) and incubated for 48 h at 37 °C. Dark gray to black colonies with halo zones were identified as characteristic coagulase-positive *S. aureus* (CPS) colonies, following Wang et al. [27].

The suspected colonies were subsequently sub-cultured onto blood agar to evaluate hemolytic activity, with β-hemolysis indicating the presence of CPS. Confirmation was achieved through the tube coagulase test, where a positive result was indicated by clot formation after incubation at 37 °C for 4 h. Further identification of typical *S. aureus* colonies was conducted using the bioMérieux VITEK^®^ 2 automated system (VITEK^®^ 2-GPID card; Marcy l’Etoile, France).

### 2.2. Antimicrobial Susceptibility Test

An antimicrobial susceptibility test (AST) assay was performed using the bioMérieux VITEK^®^ 2 automated system (AST-GP71; Marcy l’Etoile, France) by exploring the resistance of the selected CPS strains to twelve antimicrobial drugs from different classes (Table S1) following Clinical Laboratory Standards Institute (CLSI) criteria [28]. MDR was defined as resistance to three or more antibiotic families, extensive MDR was defined as resistance (XDR) to at least five families, and pan-drug resistance (PDR) was defined as resistance to all tested antibiotics [29]. For each isolate, the multiple antibiotic resistance (MAR) index was determined following Sandhu et al. [30].

### 2.3. Molecular Characterization of the Selected Strains

Molecular characterization of the *S. aureus* strains was conducted by targeting the *16S rRNA* gene. DNA extraction was carried out utilizing the TaKaRa MiniBEST Bacterial Genomic Extraction Kit Ver. 3.0 (TakaRa, Kyoto, Japan). PCR amplification was carried out with primers 27 F and 1492 R [31], using a Bio-Rad S1000™ Thermal Cycler (Bio-Rad, Hercules, CA, USA), with conditions detailed in Table S2. Amplification products were visualized on a 1.2% agarose gel stained with ethidium bromide (EtBr) under the UV light WiseDoc^®^ UV illumination system. Sequencing was conducted by Sangon Biotech (Shanghai, China), and sequence alignment was performed using the NCBI BLAST web tool (latest version as of September 2024) against the GenBank database (http://www.ncbi.nlm.nih.gov/BLAST/ (accessed on 4 September 2024)). Additionally, antimicrobial resistance and virulence genes in the *S. aureus* isolates were identified using simplex PCR, providing critical insights into the genetic determinants of resistance and pathogenicity. Six resistance genes were targeted: *blaZ* (penicillin), *aadE* and *aacA-aphD* (aminoglycoside), *apm* (apramycin), *cfr* (chloramphenicol/florfenicol), *spc* (spectinomycin), and *mecA* (methicillin). Virulence genes screened included *clfA* (clumping factor A), *fnbA* and *fnbB* (fibronectin-binding proteins), *sea*, *seb*, *sec*, *sed*, and *see* (staphylococcal enterotoxins), *tst* (toxic shock syndrome toxin), and *pvl* (Panton–Valentine leucocidin). Primer sequences are listed in Table S3 [32,33,34,35,36,37,38,39,40,41].

### 2.4. Biosynthesis of Zinc Oxide Nanoparticles

ZnO-NPs were eco-friendly and biosynthesized using the plant-mediated green synthesis method, as shown in Figure 1, following the method described by Guirguis et al. [22]. Zinc acetate was the precursor material, and *S. rebaudiana* ground leaves were used as a reducing and stabilizer agent. *S. rebaudiana* extraction was carried out based on the method described by Essa et al. [42]. A weight of five grams of ground *S. rebaudiana* leaves was suspended into 100 mL of dH_2_O for 15 min at 25 °C in an ultrasonicator (KQ3200DE, Kunshan Ultrasonic Instruments Co., Ltd., Kunshan, China), then filtered using a membrane filter (0.45 µm). The filtrate was dialyzed for 12 h in a dialysis bag and then centrifuged for 10 min at −10 °C and 6000 rpm. The obtained supernatant was mixed with ethanol in a 1:3 (*v*/*v*) ratio and subjected to centrifugation under the same conditions. The final supernatant, containing purified bioactive compounds, was collected. The synthesis of ZnO-NPs was accomplished by preparing a 0.1 M zinc acetate solution in 100 mL of deionized water, which was heated to 35 °C under continuous stirring at 500 rpm. The *S. rebaudiana* extract was then combined with the zinc acetate solution at a volume ratio of 5:3. The pH of the resulting mixture was adjusted to 10 using NaOH solution (4 M). The reaction mixture was maintained at 70 °C with constant stirring at 500 rpm for 3 h. Following the reaction period, the suspension was centrifuged to separate the precipitate and the resulting precipitate was subjected to multiple washing cycles to remove residual impurities. The purified precipitate was dried at 25 °C and pulverized into a fine powder for further applications [22].

### 2.5. Characterization of Zinc Oxide Nanoparticles

The optical characteristics of ZnO-NPs were analyzed using a UV–visible spectrophotometer (Shimadzu UV-1800, Shimadzu, Kyoto, Japan) at 300–600 nm. Functional groups were identified using a Perkin Elmer RXI FT-IR spectrophotometer, spanning 400 to 4000 cm^−1^. Transmission electron microscopy (TEM) was performed with H-800 TEM (Hitachi, Tokyo, Japan) at 100 kV. The ZnO-NP diameter and polydispersity index (PDI) were determined using dynamic light scattering (DLS) analysis with a Zetasizer Nano ZS90 (Malvern, UK) [43]. The crystallographic characterization was performed using X-ray diffraction measurements on a Miniflex system (Rigaku, Tokyo, Japan), equipped with a copper source emitting Kα radiation. The instrument parameters were set to a 40 kV voltage and 20 mA current, while data were collected at a rate of 5 degrees per min along the 2θ axis.

### 2.6. Assessment of the Bioactivity Efficacy of ZnO-NPs Against Virulent MDR S. aureus

#### 2.6.1. Antibacterial Activity

The antibacterial potential of ZnO-NPs at varying concentrations (5, 10, 15, and 20 μg) against MDR *S. aureus* strains was assessed with a disk diffusion assay following Atapakala et al. [44]. Bacterial cultures incubated for 1 day were adjusted to 0.5 McFarland standard (~1.5 × 10^8^ CFU/mL) in nutrient broth (NB) and nutrient agar (NA) plates. Sterile disks of filter paper were impregnated with ZnO-NP suspensions, placed on the agar surface, and incubated at 37 °C for 2 days. The diameter of the inhibition zones (IZ) was measured in millimeters, with the results averaged from triplicate experiments. The MIC was assessed with a serial dilution assay [22], and the ZnO-NP suspensions were inoculated to a 96-well plate containing bacterial inoculum and kept for 1 day at 37 °C. The MIC was detected as the minimum ZnO-NP concentration that completely prevents bacterial growth.

#### 2.6.2. Antibiofilm Activity

The antibiofilm efficiency of ZnO-NPs was detected using the method described by Mohanta et al. [45]. Briefly, a bacterial inoculum, diluted to OD 1.0 at 600 nm, was mixed with ZnO-NP suspensions (50, 100, 150, 200, 250 μg) in a 96-well microtiter plate [46]. To evaluate biofilm inhibition, bacterial cultures were incubated at 37 °C, and non-adherent cells were removed following 24 h of treatment. The wells were washed and fixed with sodium acetate (2%) before being stained with 0.1% crystal violet. Excess stain was removed, and the plates were dried. Biofilm inhibition was detected by the following equation:

$$\mathrm{Biofilm inhibition}\;(\%)\;=\;\frac{(ODcontrol-ODsample)\times 100}{ODcontrol}$$

### 2.7. ZnO-NPs Mechanism as Antibacterial Agent

#### 2.7.1. Cell Membrane Integrity

The damage to the bacterial cell membrane after treatment with ZnO-NPs was assessed by evaluating the release of cytoplasmic content (e.g., proteins and nucleic acids) [47]. For cell viability and protein/nucleic acid release assays, bacterial cultures were grown at 37 °C and adjusted to an OD_600_ of 1.0. *S. aureus* cells from a 100 mL suspension were collected by centrifugation at 5000 rpm for 15 min. The cells were washed three times and re-suspended in 0.1 MPBS (pH 7.4). The 100 mL washed suspension was shaken and incubated at 37 °C for 4 h in the presence of ZnO-NPs at the MIC value. After incubation, the suspension was centrifuged at 8000 rpm for 5 min. The supernatants were collected and diluted with PBS, and the absorbance at 260 nm was measured using a UV–vis spectrophotometer (Infinite 200 PRO, Grodig, Austria). A correction was performed by measuring the absorbance of PBS containing the same concentration of ZnO-NPs after 2 min of reaction with the tested strains. Untreated bacterial cells (control) were also corrected using PBS. Results were expressed in terms of the optical density of 260 nm absorbing materials at each interval (0, 4, 8, 12, 16, 20, and 24 h). Additionally, suspensions were collected to determine protein concentrations at 280 nm. The untreated sample served as a control, and phosphate-buffered saline (PBS) was used as a blank.

#### 2.7.2. Changes in Bacterial Cell Morphology

The morphological alteration in bacteria following ZnO-NPs treatment was detected by scanning electron microscopy (SEM). A culture of MDR *S. aureus* was adjusted to an optical density of 1.0 at OD_600_ nm, then treated with ZnO-NPs at their Minimum Bactericidal Concentration (MBC). The bacterial–NP mixture was incubated at 37 °C for 12 h, followed by centrifugation at 8000 rpm for 10 min. The resulting cell pellets were washed with 0.1 M phosphate buffer (pH 7.4) and fixed in glutaraldehyde (2.5%), initially at 4 °C for 1 h and then at 25 °C for 3 h. After further centrifugation, the cells were dehydrated through a graded ethanol series (30% to 100%) and stored in 100% ethanol. The dehydrated cells were sputter-coated with gold and examined under SEM (JEOL-IT500A, Tokyo, Japan).

#### 2.7.3. Changes in Bacterial DNA Content

The inhibitory effect of ZnO-NPs on bacterial DNA content was assessed using laser scanning confocal microscopy (LSCM; TCS SP5 II, Leica, Germany). Following a 24 h incubation at 37 °C and 180 rpm, the bacterial suspension treated with ZnO-NPs was combined with an equal volume of a 4′,6-diamidino-2-phenylindole (DAPI) solution (10 µg/mL). A drop of this mixture was placed on a microscope slide and incubated in the dark for 10 min. A control sample, prepared without ZnO-NPs, was also included for comparison. The DAPI-stained DNA was visualized at 364 nm and 454 nm [48].

### 2.8. Data Analysis

Data were analyzed with the GraphPad Prism version 8.0.2. However, hierarchical cluster analysis was performed using PC-ORD (v. 7) with Sorensen techniques for group linkage. ANOVA was applied for multiple comparisons at *p* < 0.05 and Tukey’s test for post hoc analysis. Pearson’s correlation coefficient was applied to determine correlations.

## 3. Results and Discussion

### 3.1. Prevalence of S. aureus in Fish Samples

Of the 120 fish samples collected from local markets in Zagazig City, Egypt, *S. aureus* contamination was detected in 54.2% (65 samples). Contamination rates varied among fish species, with Nile tilapia showing 11.7% (14/120) contamination, European sardine exhibiting the highest rate at 27.5% (33/120), and Atlantic mackerel at 15% (18/120). Among the 387 suspected *S. aureus* isolates, 171 were confirmed as coagulase-positive *S. aureus* (CPS). The differences in *S. aureus* contamination rates across fish species suggest that factors such as handling, storage conditions, and environmental influences may contribute to susceptibility [49]. The high contamination rate in European sardine indicates the necessity for further investigation into its handling and sale practices to ensure compliance with food safety standards, and enhanced hygiene protocols in local markets could help mitigate *S. aureus* contamination in fish. Comparative studies from other regions reveal variations in *S. aureus* contamination rates. In India, Kumar et al. [49] reported a 15.78% contamination rate in fresh seafood samples, while Sivaraman et al. [50] found 13.65% contamination in fish and fish products. In Egypt, Mohamed and Thabet [51] found *S. aureus* contamination in 32% of 50 fish samples. Variations in reported contamination rates can be attributed to differences in sample sizes, geographical locations, fish species, and detection methodologies.

Antibiotic resistance profiling of the 171 *S. aureus* strains tested against 12 commercial antibiotics revealed significant levels of resistance (Table S4). Among the strains, 137 (80.1%) were multidrug-resistant (MDR), 105 (61.4%) were extensively drug-resistant (XDR), and 12 (7%) were pan-drug resistant (PDR). The highest susceptibility was observed against cefotaxime (66.1%), chloramphenicol (64.9%), and enrofloxacin (61.4%). The lowest efficacy was observed for tetracycline, polymyxin, marbofloxacin, amikacin, and gentamicin, with sensitivity levels ranging from 46.8% to 24.6%. The multiple antibiotic resistance (MAR) index of the *S. aureus* strains ranged from 0.25 to 1.0, with an average of 0.57, suggesting exposure to high-risk environments with frequent antibiotic use [4]. A total of 24 distinct drug resistance patterns (DRPs) were identified among the MDR *S. aureus* isolates (Table S5), with the most prevalent patterns including P4, P4a, P4b, P5, P5a, P5c, P6, P6a, P7a, P8, and P10, which collectively accounted for 74 isolates (54% of MDR strains). To further examine the genetic characteristics of these resistant strains, one MDR *S. aureus* strain was randomly selected from each of the 24 DRPs for molecular characterization.

The incidence of MDR *S. aureus* in this study was higher than that reported by Kukułowicz et al. [52], who found 59% contamination in 44 fish samples, with fish being 25% more susceptible to *S. aureus* than crustaceans. In contrast, Murugadas et al. [53] found lower resistance rates in Kerala, India, where 13.8% of fish, 9.3% of crustaceans, and 12% of mollusk samples tested positive for antibiotic-resistant *S. aureus*. Kumar et al. [49] emphasized the rising threat to public health due to the misuse of antibiotics in aquaculture, which has contributed to the increasing prevalence of *S. aureus* in fresh fish, ready-to-eat (RTE) seafood, seafood-processing plants, and among food handlers. The high prevalence of *S. aureus* contamination in fish samples, coupled with extensive antibiotic resistance, raises serious food safety concerns. The study underscores the urgent need for routine bacterial surveillance in retail fish markets, along with strict hygiene measures, improved regulatory inspections, and targeted intervention strategies to control *S. aureus* contamination.

This study, investigating antibiotic resistance genes in 24 MDR *S. aureus* strains, provides significant insights into the genetic mechanisms contributing to antibiotic resistance and pathogenicity. The findings reveal a high prevalence of resistance genes. Among these, 63% of the strains harbor *bla*Z and *aac*A-*aph*D genes, conferring resistance to penicillin and aminoglycosides, respectively. Additionally, *mec*A (58%) and *apm* (54%) genes were identified, indicating resistance to methicillin and apramycin. The *cfr* (46%) and *spc* (42%) genes were also prevalent, while *aad*E (38%) contributed to further aminoglycoside resistance. This widespread resistance highlights the adaptability of *S. aureus* in evading commonly used antibiotics, complicating treatment approaches. The study also identifies virulent factors, with 63% of the strains carrying the *fnb*B gene linked to fibronectin-binding proteins, which are critical for adhesion. Conversely, only 29% carried the *fnb*A gene. Additionally, 54% of strains harbored the *pvl* and *clf*A genes, while enterotoxin genes *sed* and *see* were present in similar proportions. The presence of *sea*, *seb*, *sec*, and *tst* genes ranged from 33% to 50%, underscoring the pathogenic potential of these strains and the importance of continuous monitoring.

A deeper analysis of the key resistance genes emphasizes their role in *S. aureus* survival. The *bla*Z gene encodes beta-lactamase, conferring penicillin resistance, while *aac*A-*aph*D enables resistance to aminoglycosides, limiting treatment options [54]. The *mec*A gene plays a critical role in methicillin resistance, encoding penicillin-binding protein 2a (PBP2a), which reduces beta-lactam binding affinity and contributes to methicillin-resistant *S. aureus* (MRSA) emergence [55]. The *apm* gene, conferring apramycin resistance, adds another layer of complexity to treatment [56]. The *cfr* gene, associated with chloramphenicol and florfenicol resistance, exemplifies *S. aureus’*s genetic plasticity, particularly in horizontal gene transfer, exacerbating the spread of resistance traits in bacterial populations [57]. The *fnb*B and *fnb*A genes, encoding fibronectin-binding proteins, facilitate bacterial adhesion, a critical step in infection establishment [58]. The *pvl* gene encodes Panton–Valentine leucocidin, a potent toxin that targets leukocytes, contributing to severe tissue infections [59]. This study further identifies several exotoxin genes (*sed*, *see*, *sea*, *seb*, *sec*, and *tst*), which enhance virulence by promoting toxin-mediated immune evasion and superantigen activity, leading to conditions like toxic shock syndrome [56,59]. The obtained results underscore the urgent need for improved antibiotic stewardship, continuous resistance monitoring, and novel therapeutic approaches.

**Figure 2:**
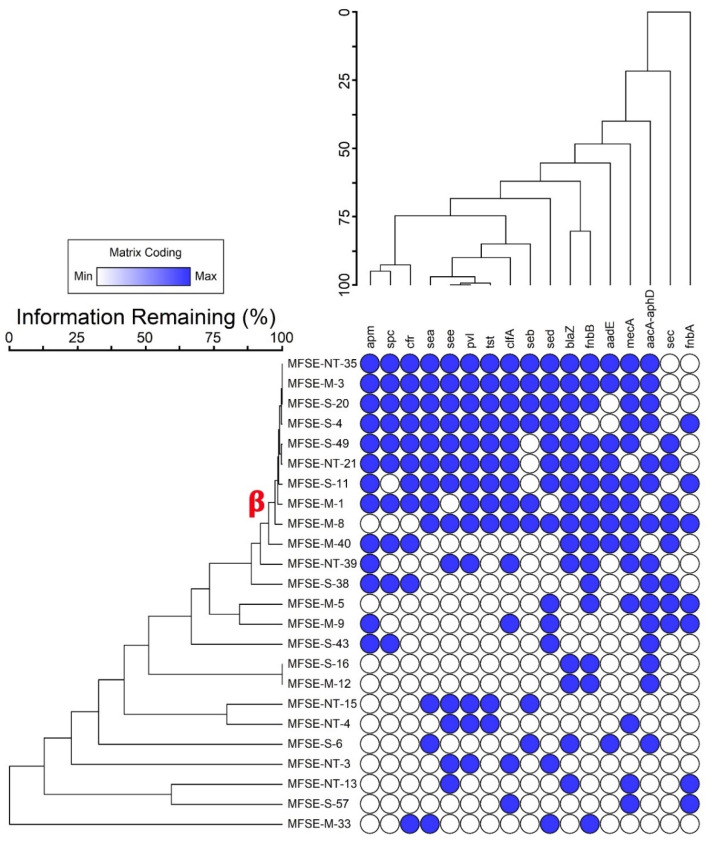
Two-way dendrogram clustering analysis of virulent MDR *S. aureus* strains using Sorensen methods. *apm*; apramycin-resistance gene, *blaZ*; penicillin resistance, *cfr*; chloramphenicol/florfenicol resistance, *tst*; toxic shock syndrome toxin, *mecA*; methicillin resistance, *spc*; spectinomycin resistance, *aad*E and *aac*A-*aph*D; aminoglycoside resistance, *sea*, *seb*, *sec*, *sed*, *see*; staphylococcal enterotoxins, *pvl*; Panton–Valentine leucocidin, *fnbA* and *fnbB*; fibronectin-binding proteins, and *clfA*; clumping factor A.

The molecular identification of the selected *S. aureus* strains was carried out by *16S rRNA* gene sequencing. As shown in the phylogeny analysis in Figure S1, MFSE-M-1, MFSE-NT-21, and MFSE-S-49 showed 100% identity with *Staphylococcus aureus* 14507 (CP053356), *Staphylococcus aureus* UP_644 (CP047841), and *Staphylococcus aureus* GIMC7004:SaO-15 (CP166018), respectively. MFSE-S-11, MFSE-S-4, and MFSE-S-20 showed 99.81, 99.55, and 99.06% identity to *Staphylococcus aureus* MA22 (KX655882), *Staphylococcus aureus* HKG 299 (KY674887), and *Staphylococcus aureus* MJ163 (CP038229), respectively. However, MFSE-M-8 showed 98.69% identity to *Staphylococcus aureus* GCC_20MS (MZ305087). MFSE-M-3 and MFSE-NT-35 showed 97.78 and 97.84%, identity to *Staphylococcus aureus* CC5-MSSA (CP155060) and *Staphylococcus aureus* CLRSA3 (JQ429750), respectively. Clustering analyses revealed nine MDR *S. aureus* harboring the highest virulence and resistance genes (Cluster β), as illustrated in Figure 2.

### 3.2. Characterization of the Green-Synthesized ZnO-NPs

**Figure 3:**
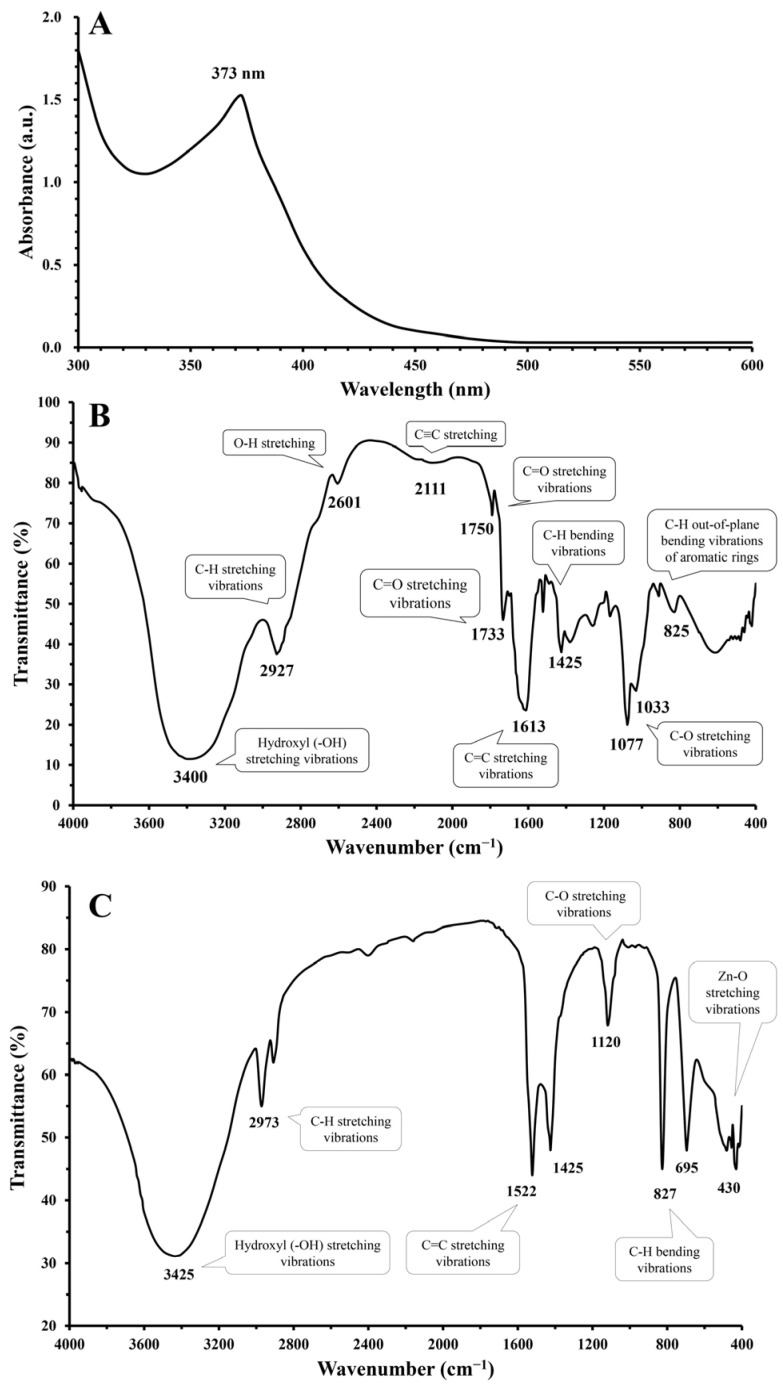
The green-synthesized ZnO-NPs characterization using UV–vis spectra (**A**), FTIR spectra analysis of *S. rebaudiana* (**B**), and FTIR spectra analysis of biosynthesized ZnO-NPs (**C**).

The green-synthesized ZnO-NPs were characterized by UV-visible spectroscopy, which identified a distinct absorption peak. As depicted in Figure 3A, the absorption peak for the ZnO-NPs was observed at a wavelength of 373 nm. This peak is characteristic of ZnO-NPs and is indicative of their unique optical properties, which are associated with a high excitation binding energy. The identification of this peak confirms the successful formation of ZnO-NPs and underscores the significant role of *S. rebaudiana* as a reducing agent in the environmentally friendly synthesis of ZnO-NPs. The 373 nm absorption peak is a signature of pure ZnO, reflecting electronic transitions within the material. In the context of green synthesis, *S. rebaudiana* provides essential reducing agents that facilitate the conversion of zinc ions into ZnO-NPs. Raghu and Velayudhannair [60] highlighted the crucial role of phytochemicals, particularly polyphenols and flavonoids found in plant extracts, in the green synthesis of nanoparticles. These biomolecules act as reducing and stabilizing agents, driving the formation of nanoparticles with controlled sizes, shapes, and properties. This eco-friendly approach, leveraging the inherent reducing power of natural resources, offers a sustainable alternative to conventional, often environmentally burdensome, nanoparticle synthesis methods. Utilizing plant extracts for nanoparticle fabrication aligns with the growing emphasis on sustainable practices within nanotechnology, minimizing the reliance on hazardous chemicals and harsh reaction conditions.

The FTIR spectrum of *S. rebaudiana* extract revealed characteristic peaks corresponding to various functional groups, confirming the presence of bioactive compounds involved in the green synthesis of ZnO-NPs. As shown in Figure 3B, the broad peak at 3400 cm^−1^ is attributed to the O-H stretching vibrations of hydroxyl (-OH) groups, which are abundant in phenolic compounds, flavonoids, and glycosides present in *S. rebaudiana* [61]. These hydroxyl groups play a crucial role as reducing and stabilizing agents in the green synthesis of ZnO-NPs. The peak at 2927 cm^−1^ corresponds to the C-H stretching vibrations of aliphatic compounds [22], while the peak at 2601 cm^−1^ is associated with O-H stretching, further supporting the presence of hydroxyl-containing biomolecules. The band observed at 2111 cm^−1^ may be related to the C≡C stretching of alkynes [62], which could be part of secondary metabolites in *S. rebaudiana*.

The presence of carbonyl functional groups is indicated by peaks at 1750 cm^−1^ and 1733 cm^−1^, which correspond to C=O stretching vibrations, likely originating from esters or carboxylic acids [63]. Additionally, the peak at 1613 cm^−1^ is attributed to the stretching vibrations of aromatic C=C bonds, confirming the presence of polyphenols [64], which are known constituents of *S. rebaudiana*. The peak at 1425 cm^−1^ is assigned to the C-H bending vibrations of alkyl groups, while the peaks at 1077 cm^−1^ and 1033 cm^−1^ correspond to the C-O stretching vibrations of carbohydrates and glycosides [65]. The presence of aromatic compounds is further confirmed by the peak at 825 cm^−1^, which is attributed to the C-H out-of-plane bending vibrations of aromatic rings [66]. Thus, the FTIR spectrum of *S. rebaudiana* extract highlights the presence of various functional groups that play a significant role in reducing, capping, and stabilizing ZnO-NPs during biosynthesis.

Furthermore, FTIR spectroscopy analysis of the green-synthesized ZnO-NPs revealed a rich array of characteristic peaks, each providing insight into the functional groups involved and confirming the successful green synthesis process (Figure 3C). The prominent peak at 3425 cm^−1^ is associated with the stretching vibrations of hydroxyl (-OH) groups, which are abundant in *S. rebaudiana* leaf extract [22,67]. These hydroxyl groups play a crucial role as capping, stabilizing, and reducing agents to stabilize and prevent ZnO-NPs agglomeration. Additionally, the observed peak at 2973 cm^−1^ is attributed to the C-H stretching vibrations of alkyl groups, which are part of the organic compounds in leaf extract. These alkyl groups likely contribute to the nanoparticles’ stability through van der Waals interactions [67]. At 1522 cm^−1^, a peak is observed that can correspond to the stretching vibrations of alkene bonds (C=C) in aromatic compounds, indicative of phenolic compounds such as stevioside and rebaudioside, which are known constituents of *S. rebaudiana* [68]. These compounds facilitate the reduction of zinc ions, aiding in ZnO-NPs formation. The peak at 1425 cm^−1^ (C-H bending vibrations of alkyl groups) further suggests the involvement of these organic components in nanoparticle stabilization.

Another significant peak at 1120 cm^−1^ is linked to the C-O stretching vibrations of alcoholic and ether groups found in the carbohydrates and glycosides of the leaf extract. These groups may interact with the ZnO-NPs surface, enhancing their stability. The presence of phenolic compounds is further confirmed by the peak at 827 cm^−1^, which corresponds to the C-H out-of-plane bending vibrations of aromatic rings, emphasizing their role in green synthesis [69]. The peak at 695 cm^−1^, attributed to the C-H out-of-plane bending vibrations of alkenes, suggests additional stabilization through π-π interactions. Crucially, the peak at 430 cm^−1^ indicates Zn-O stretching vibrations, directly confirming the formation of ZnO nanoparticles [70]. This peak, characteristic of the ZnO crystal structure, underscores the success of utilizing *S. rebaudiana* in the environmentally friendly synthesis of ZnO-NPs.

The FTIR analysis revealed modifications in the intensity and morphology of certain bands compared to the original *S. rebaudiana* extract spectrum. These alterations suggest that specific functional groups actively participate in the synthetic process. Additionally, the complete disappearance of particular bands indicates that their corresponding functional groups are directly involved in the reduction of Zn^2+^ to Zn and the subsequent formation of ZnO-NPs [22]. Moreover, FTIR analysis indicates that the hydroxyl (-OH) groups detected in ZnO-NPs originate primarily from plant-derived phytochemicals rather than retained water. The FTIR spectrum of *S. rebaudiana* extract showed a hydroxyl (-OH) peak at 3400 cm^−1^, closely aligning with the 3425 cm^−1^ peak in ZnO-NPs, suggesting a role in capping and stabilization. If residual water were the main contributor, a broader peak and additional strong absorption at 1600–1700 cm^−1^ (H-O-H bending) would be expected, which was not observed [71,72,73]. Furthermore, key functional groups from *S.* rebaudiana (e.g., 1522 cm^−1^, 1425 cm^−1^, 825 cm^−1^, 603 cm^−1^) were also present in the ZnO-NPs, reinforcing plant-based stabilization.

Therefore, *S. rebaudiana* extract can act as a reducing agent due to the presence of phytochemicals (e.g., polyphenols, flavonoids, terpenoids, and glycosides) that have reducing properties as stated in several studies [22,60,74]. These compounds donate electrons to the zinc ions (Zn^2+^) from zinc acetate, reducing them to metallic zinc (Zn^0^). The metallic zinc then reacts with oxygen (from the environment or the solution) to form ZnO-NPs. The same phytochemicals in *S. rebaudiana* extract also act as capping and stabilizing agents. They adsorb onto the surface of the newly formed ZnO-NPs, preventing their aggregation and ensuring the nanoparticles remain dispersed and stable in the solution. Basnet et al. [75] stated that the ZnO-NP formation mechanism involved zinc acetate dissociating in water to release Zn^2+^ ions and phytochemicals in *S. rebaudiana* extract to reduce Zn^2+^; to Zn^0^.

$${Zn}^{2+}+{2e}^{-}\to {Zn}^{0}$$

The metallic zinc (Zn^0^) reacts with oxygen (O_2_) or hydroxyl ions (OH^−^) in the solution to form ZnO.

$${Zn}^{2+}+\frac{1}{2}\;O_{2}\to ZnO$$

Or

$${Zn}^{0}+2\;OH\to ZnO+H_{2}O$$

**Figure 4:**
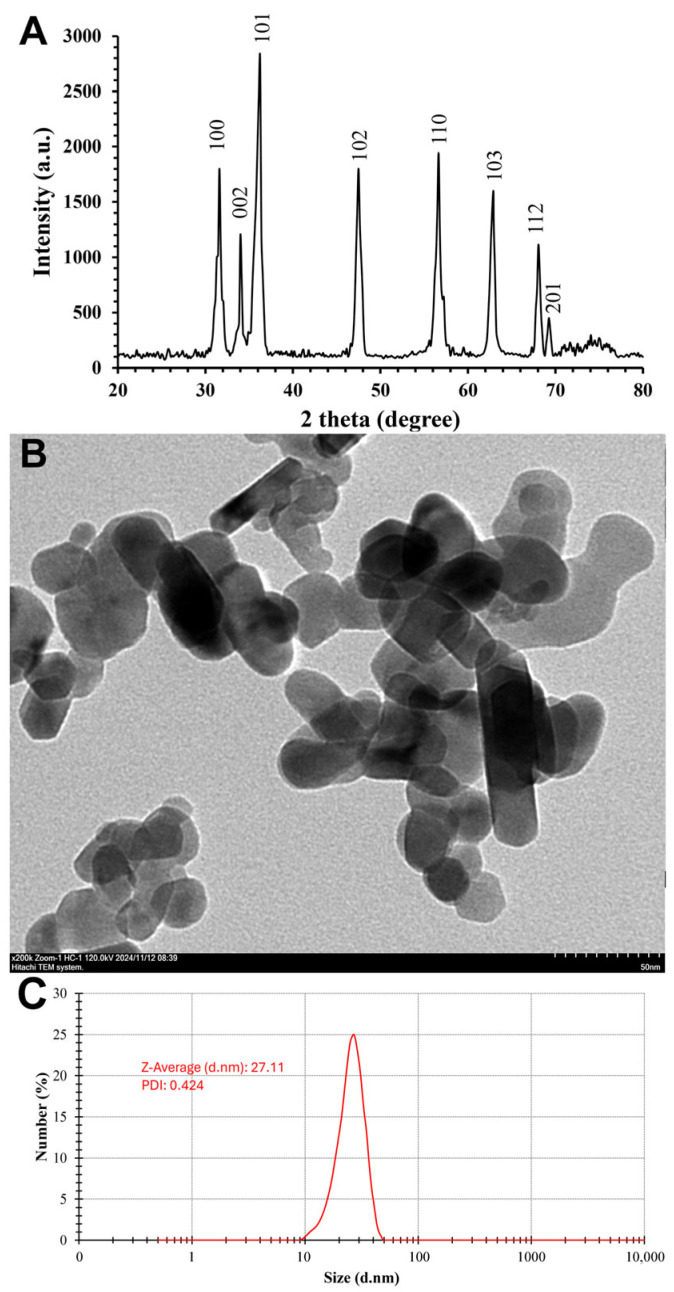
The green-synthesized ZnO-NPs characterization using X-ray diffraction analysis (**A**), transmission electron microscopy image (**B**), and dynamic light scattering analysis (**C**).

The crystalline structure of ZnO-NPs was characterized through XRD analysis, as illustrated in Figure 4A, showing distinct and sharp peaks at specific diffraction angles and indicating ZnO-NPs high purity and crystallinity, consistent with the data obtained by Tyagi et al. [76]. The analysis identified corresponding Miller indices for the crystallographic planes (100), (002), (101), (102), (110), (103), (112), and (201), with diffraction peaks observed at 31.6°, 34.04°, 36.25°, 47.52°, 56.63°, 62.9°, 68.05°, and 69.26°, respectively. The peak positions closely match the standard XRD data for hexagonal wurtzite ZnO (JCPDS No. 36-1451), proving the successful synthesis of ZnO-NPs with high structural integrity. This nanoscale particle size is significant for the material’s potential applications in catalysis, electronics, and biomedical engineering fields. The results provide valuable insights into the structural characteristics of ZnO-NPs, demonstrating their successful synthesis through eco-friendly methods and reinforcing their suitability for advanced technological applications.

As shown in Figure 4B, TEM analysis was utilized to confirm the successful synthesis of ZnO-NPs. The analysis revealed that ZnO-NPs exhibit quasi-spherical, rod, hexagonal, and irregular morphologies. The DLS analysis indicated the hydrodynamic diameter of the produced nanoparticles, with a Z-average size of 27.11 nm and PDI of 0.424, as shown in Figure 4C. The results also suggest that capping agents play a crucial role in stabilizing the nanoparticles and may influence their interactions in various biological applications. Furthermore, the negatively charged surface of the green-synthesized ZnO-NPs, as reported by Yassin et al. [77], indicates that electrical repulsion between particles enhances their stability.

The synthesis of ZnO-NPs in diverse configurations is largely dependent on the role of capping agents. A deeper understanding of the particle formation process is essential for controlling the shape of ZnO-NPs effectively. Notably, *S. rebaudiana* has shown promise as a biocatalyst for the complexation of zinc ions (Zn^2+^), due to its rich composition of carbohydrates, amines, hydroxyl groups, vitamins, and proteins. The unique biochemical properties of *S. rebaudiana* facilitate the formation of stable complexes with zinc, thereby preventing the aggregation of zinc species. This complexation process is especially important as it enhances the solubility and bioavailability of zinc, making it more accessible for use in various applications [77].

### 3.3. ZnO-NPs Antibacterial and Antibiofilm Efficacy

The antibacterial effect of ZnO-NPs was explored against pathogenic MDR *S. aureus* strains using a disk diffusion technique. The MDR *S. aureus* strains selected for this assay represent a significant challenge for conventional antimicrobial treatments due to their harboring of highly virulent and resistant genes. Based on the results in Table 1, the IZ increases with the concentration of ZnO-NPs for most isolates, indicating a dose-dependent antibacterial effect. This is evident as the IZ is generally larger at higher concentrations (20 µg/disk) compared to lower concentrations (5 µg/disk). MFSE-M-1 shows a significant increase in the IZ from 5 µg/disk (10.9 ± 0.4 mm) to 20 µg/disk (20.1 ± 0.4 mm). The differences are statistically significant (*p* < 0.05). For other isolates like MFSE-NT-21, MFSE-S-49, and MFSE-S-11, there are significant differences between some concentrations (e.g., 5 µg/disk vs. 20 µg/disk) with lower variation in the significant differences of their IZ across concentrations. MFSE-S-11, MFSE-S-20, MFSE-M-3, and MFSE-NT-35 show statistically significant differences in IZ ranging from 23.5 ± 1.0 to 24.4 ± 0.4 at different concentrations, with the highest inhibition observed at 20 µg/disk. The high *p*-values (0.858, 0.951, 0.861, 0.985) across the different concentrations indicate that the differences in inhibition zones among the concentrations are not statistically significant. This suggests that while there is a general trend of increasing inhibition with higher ZnO-NP concentrations, the variation might not be substantial enough to be statistically significant for all isolates. The obtained data were consistent with El-Masry et al. [78] who found that the antibacterial properties of ZnO-NPs with an average size of 20 nm towards enterotoxigenic *S. aureus* were investigated in vitro and resulted in IZ ranging from 22 mm to 26 mm.

The green-synthesized ZnO-NPs exhibited potent antibacterial activity against a panel of multidrug-resistant *S. aureus* strains, as evidenced by their low MIC and MBC values. MIC values ranged from 6.25 µg/mL to 25 µg/mL, while MBC values were between 12.5 µg/mL and 50 µg/mL. Notably, strains MFSE-S-11, MFSE-S-4, MFSE-S-20, and MFSE-NT-35 (isolated from European sardine and Nile tilapia) exhibited the highest susceptibility, with an MIC of 6.25 µg/mL. MFSE-NT-21 strain (isolated from Nile tilapia) displayed a comparatively higher MIC of 25 µg/mL. This observed variability in MIC and MBC values across strains likely reflects inherent differences in resistance mechanisms, potentially attributed to variations in genetic makeup and virulence factors. These findings align with previous research by Ahmad et al. [79], who similarly reported the significant antibacterial efficacy of ZnO-NPs against various virulent bacterial strains. Notably, Ahmad et al. observed inhibition zones ranging from 16 to 21 mm, with MIC values from 15.6 to 125 µg/mL and MBC values from 62.5 to 250 µg/mL. This collective evidence underscores the potential of green-synthesized ZnO-NPs as promising antimicrobial agents for mitigating the risks of multidrug-resistant bacterial pathogens in food production and aquaculture, ultimately contributing to improved food safety.

Regarding the antibiofilm activity, the obtained data showed that ZnO-NPs have a high biofilm inhibition potential against resistance and virulence gene-producing *S. aureus*. Table 2 reveals the concentration-dependent inhibition of biofilm formation and the significant biofilm inhibition increase with increasing concentrations of ZnO-NPs. Table 2 shows that the MFSE-M-3 strain showed maximum inhibition at 250 µg (92.1%), suggesting higher susceptibility to ZnO-NPs. In contrast, the MFSE-S-11 strain was more resistant (72.2%) at 250 µg. Also, a statistical analysis using a *t*-test, as depicted in Figure S2, showed significant variation (*p* = 0.0114) between the susceptibility of tested strains at 200 µg and 250 µg.

Additionally, significant variation (*p* < 0.0001) was observed between the susceptibility of MDR *S. aureus* strains biofilm at 150 µg and 200 µg (Figure S2). ZnO-NPs demonstrate strong and concentration-dependent antibiofilm activity against virulent MDR *S. aureus* strains. Higher concentrations (200 µg/mL and 250 µg/mL) are generally more effective, achieving 80–90% inhibition for most isolates. However, the response to ZnO-NPs varies between isolates, potentially due to differences in genetic makeup or biofilm-forming capabilities. The lack of significant differences according to ANOVA single-factor analysis between the isolates at each concentration suggests that ZnO-NPs are broadly effective against different strains, highlighting their potential application to mitigate biofilm-related challenges in food safety and public health.

Biofilm comprises complex communities of bacteria enclosed in an extracellular polymeric substance (EPS), which enhances their resistance to conventional antibiotics [80]. The EPS matrix can be a protective barrier, shielding bacteria from antimicrobial agents by impeding their penetration and preventing adhesion to bacterial cell walls [81]. ZnO-NPs possess unique properties, such as a high surface area-to-volume ratio and potent antibacterial activity, which allows them to disrupt biofilm architecture. This makes them highly suitable for medical and industrial applications [8].

The biofilm inhibition capacity of ZnO-NPs increases with concentration. Sub-MIC levels can reduce biofilm formation by over 60%, primarily by degrading the EPS matrix and weakening biofilm integrity [80]. ZnO-NPs can also inhibit EPS production, directly impeding bacterial growth [44]. Their effectiveness varies based on environmental conditions, with greater efficacy in nutrient-poor environments, making them particularly useful in clinical settings where biofilms form on medical devices and implants [81]. Coating such devices with ZnO-NPs reduces bacterial colonization, lowering the risk of biofilm-related infections, particularly from antibiotic-resistant bacteria [82]. Additionally, ZnO-NPs have the potential in food packaging to inhibit bacterial growth, ensuring food safety and extending shelf life [80].

In terms of safety and ZnO-NPs applicability, the Overall Migration Level (OML) of ZnO-NPs is a key parameter for assessing their inertness in food-contact applications. According to regulatory guidelines, ZnO-NPs incorporated into polymers must comply with the migration limit of 60 mg/kg of food. Additionally, the European Plastics Regulation (EU 10/2011) and Commission Regulation (EU 2016/1416) have established a Specific Migration Limit (SML) of 5–25 mg of zinc per kg of food for food-contact materials. Further reinforcing safety considerations, the National Institute of Health (NIH) has set a daily zinc consumption limit of 40 mg for the human body [18]. Compliance with these regulations ensures that ZnO-NPs used in food packaging remain within acceptable safety thresholds. Experimental studies have further validated the safety of ZnO-NPs in food applications. For instance, Bumbudsanpharoke and Ko [83] examined the migration of Zn^2+^ ions from LDPE-ZnO nanocomposite films, reporting a migration level of 3.5 mg/L, which falls below the European Plastics Regulation’s migration limit and is considered non-toxic to human health. Additionally, Aristizabal-Gil et al. [84] investigated alginate nanocomposites containing ZnO-NPs and ZnO/CaO-NPs. They found that while a concentration of 5 g/L exceeded the maximum migration limit, a lower concentration of 0.5 g/L ZnO-NPs remained within the approved migration threshold. Moreover, Heydari-Majd et al. [85] studied ZnO-NP migration in fish filets wrapped with PLA bionanocomposite films. Their findings showed a slight increase in migrated Zn^2+^ levels over storage time, particularly when essential oils were incorporated into the nanocomposite. However, despite this increase, the Zn^2+^ migration remained below the maximum limit set by the NIH, ensuring its safety for food applications. These findings confirm that ZnO-NPs, when used within regulatory limits and proper formulations, pose minimal risk to human health while effectively enhancing food preservation and safety.

ZnO-NPs have also shown promise in inhibiting biofilm formation by MDR *S. aureus* strains isolated from fish products. ZnO-NPs can inhibit biofilm formation by up to 76% at sub-MIC concentrations of 1024 µg/mL by interfering with biofilm-related genes such as *icaA*, *icaD*, and *fnbA* [86]. Furthermore, ZnO-NPs reduce bacterial cell surface hydrophobicity, diminishing bacterial adherence to surfaces and preventing biofilm formation [46]. ZnO-NPs also reduce the hemolytic activity of *S. aureus* by downregulating virulence-related genes, highlighting their potential as antivirulence agents [46]. Their ability to accelerate wound healing in infections caused by *S. aureus* suggests further potential for clinical applications. Consequently, the antibiofilm activity of ZnO-NPs, especially against MDR *S. aureus*, presents a promising strategy for combating biofilm-related infections. By disrupting biofilm formation, reducing virulence, and enhancing antimicrobial efficacy, ZnO-NPs offer a valuable tool in the fight against antibiotic-resistant bacterial infections. Further studies are needed to explore their full potential in clinical applications.

### 3.4. ZnO-NPs Mode of Action as Antibacterial Agents

The capability of ZnO-NPs to compromise cell membrane integrity was estimated by quantifying the release of intracellular constituents (e.g., proteins and nucleic acids) into the medium. To evaluate the extent of membrane disruption, the study quantified the release of these cellular macromolecules by monitoring the absorbance of the supernatant at 260 nm and 280 nm, which correspond to the absorption maxima of nucleic acids and proteins, respectively.

**Figure 5:**
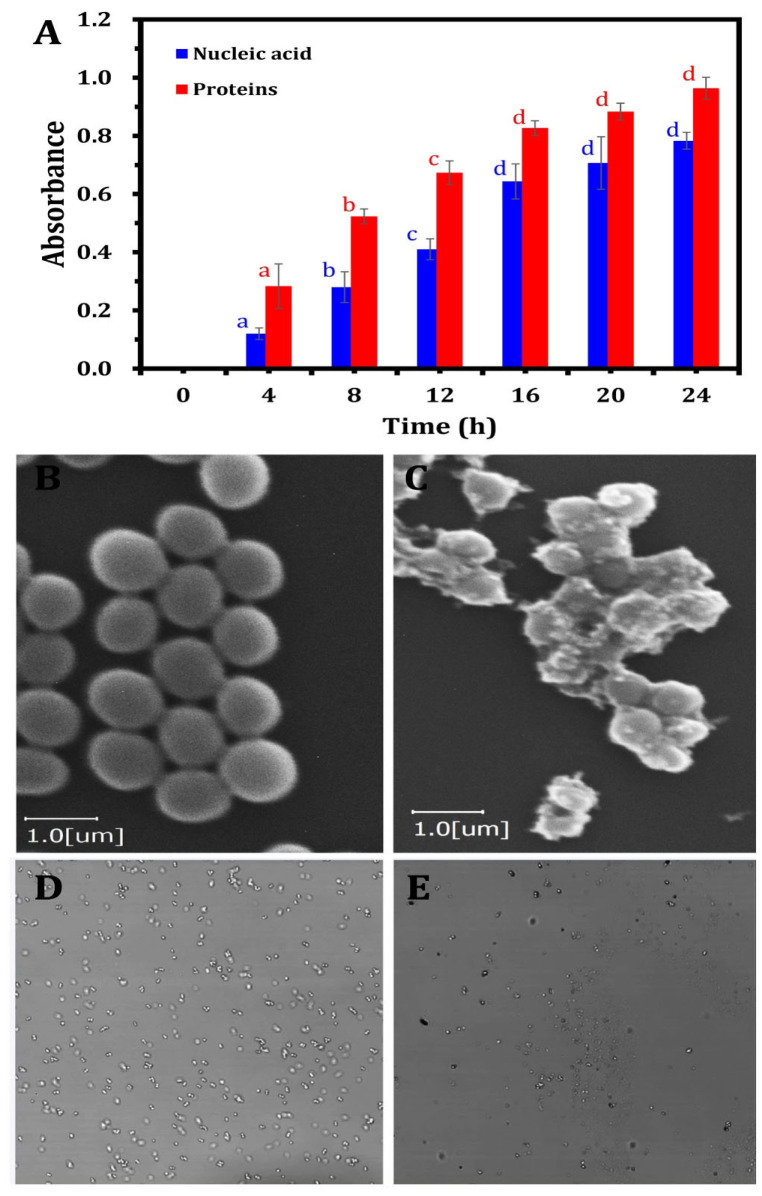
The effect of ZnO-NPs towards pathogenic MDR *S. aureus* cells. The releasing of intracellular components (nucleic acid and proteins) after treatment using ZO-NPs (**A**); scanning electron microscope micrographs of *S. aureus* cells without treatment (**B**) and after treatment by ZO-NPs (**C**); alteration in bacterial DNA content by LSCM for *S. aureus* cells showing DNA before treatment (**D**) and after ZnO-NP treatment (**E**).

As depicted in Figure 5, the supernatant from ZnO-NP-treated samples showed a marked increase in absorbance at 260 nm and 280 nm compared to the control group. This observation provides compelling evidence that ZnO-NPs compromise the structural integrity of bacterial cell membranes, leading to a substantial release of proteins and nucleic acids into the medium. The leakage of these vital intracellular components indicates a disruption of the bacterial membrane’s barrier function, resulting in the uncontrolled efflux of essential macromolecules. This disruption of membrane integrity underscores the potent antimicrobial activity of ZnO-NPs against the MDR *S. aureus* strain tested in this study. The increased absorbance, relative to the control, reflects the extent of membrane damage caused by ZnO-NPs, confirming their disruptive effect on bacterial cell integrity. Damage to the bacterial cell membrane plays a crucial role in determining the ability of bacteria to sustain normal growth. The observed release of cellular components, including proteins and nucleic acids, increased progressively with prolonged exposure to ZnO-NPs. Statistical analysis (Figure 5A) revealed that the release of these biomolecules significantly increased (*p* < 0.05) up to 12 h of incubation, followed by a non-significant increase until 24 h. Similarly, Yang et al. [87] observed that the permeability of bacterial cell membranes was enhanced when nanoparticles infiltrated bacterial cells, primarily due to lipid layer damage mediated by ROS.

The current study demonstrates that ZnO-NPs exhibit significant antibacterial activity by disrupting the membrane integrity of MDR *S. aureus*. Treated bacterial cells displayed marked changes in dispersion and cellular size, contrasting with the uniformly smooth surface morphology of untreated cells (Figure 5B). To further elucidate the external morphological alterations induced by ZnO-NPs at the MBC, SEM analyses were performed (Figure 5C). The SEM imaging demonstrated that after 24 h of ZnO-NP treatment at their respective MBCs, bacterial cells exhibited irregular surface folding, indicative of severe morphological alterations. This structural disruption was accompanied by substantial cellular damage, characterized by cell aggregation and fragmentation. These findings provide strong evidence of the extensive morphological and structural changes induced in bacterial cells following exposure to ZnO-NPs. Collectively, these findings suggest that ZnO-NP treatment compromises the permeability and integrity of the bacterial membrane, resulting in extensive membrane damage in MDR *S. aureus* cells.

The LSCM analysis demonstrated significant variations in bacterial DNA content within *MDR S. aureus* cells following treatment with ZnO-NPs compared to the control groups. ZnO-NPs exerted a pronounced effect on DNA quantity, as evidenced by the higher bacterial DNA incidence observed in the untreated control sample (Figure 5D) relative to the treated bacteria. The interaction between the nano–bio hybrid system and bacterial DNA may lead to DNA damage, strand breakage, or alterations in DNA packaging (Figure 5E). These molecular disruptions can interfere with fundamental biological processes such as DNA replication, transcription, and repair, ultimately inhibiting bacterial proliferation or inducing cell death.

Consistent with these findings, Elabbasy et al. [26] reported that antibacterial agents compromise cytoplasmic membrane permeability, disrupting both DNA integrity and ATP biosynthesis, thereby leading to microbial cell death. Similarly, Mendes et al. [88] identified increased bacterial cell wall permeability as a key mechanism through which ZnO-NPs exert their antibacterial effects. This alteration allows for the influx of DNA and proteins, which can disrupt essential cellular processes. The increased permeability is likely because of the interaction of ZnO-NPs with the lipid bilayer of the bacterial cell membrane, leading to structural destabilization [89]. The study observed significant changes in the morphology of the bacterial cells upon treatment with ZnO-NPs. SEM images revealed irregularities in the cell surface, including folding and distortion. These morphological alterations indicate membrane damage, which can result in the loss of structural integrity and functionality of the bacterial cell.

**Figure 6:**
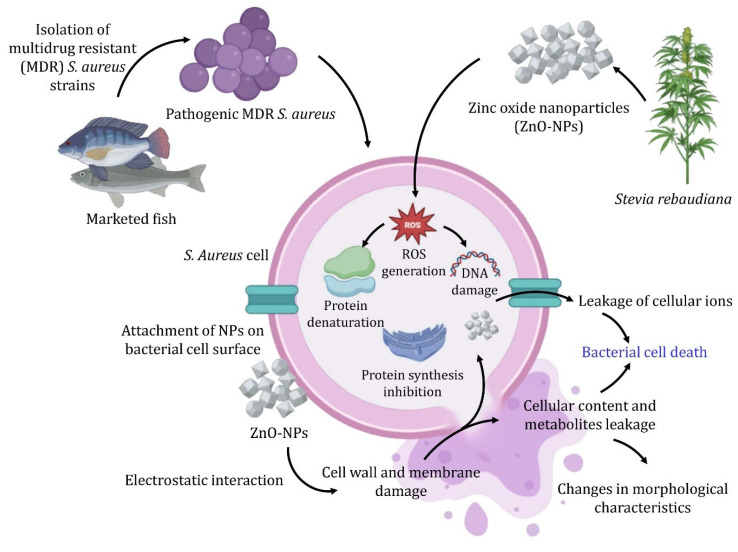
The proposed mechanism for the antibacterial activity of zinc oxide nanoparticles against *S. aureus* bacterial cell.

Li et al. [90] demonstrated that ZnO-NPs generate ROS leading to oxidative stress within bacterial cells, causing lipid peroxidation, protein denaturation, and DNA damage, ultimately resulting in cell death. As shown in Figure 6, the ROS generated by ZnO-NPs damage cellular components and disrupt the extracellular matrix of biofilms, facilitating the penetration of antibacterial agents [86,91]. In addition to ROS production, ZnO-NPs enhance their antibacterial efficacy by releasing zinc ions (Zn^2+^) in aqueous environments. These ions interfere with bacterial metabolic pathways, inhibit essential enzymes, and damage cell membranes, often leading to cell lysis. ZnO-NPs have demonstrated effectiveness against MDR *S. aureus*. Moreover, ZnO-NPs inhibit biofilm formation by compromising the biofilm matrix’s structural integrity, thus increasing bacterial susceptibility to antibiotics and other antimicrobials.

The positive surface charge of ZnO-NPs enhances their electrostatic interaction with the negatively charged bacterial cell wall, promoting strong adhesion. This results in increased nanoparticle concentrations at the bacterial surface, leading to physical disruption of the cell wall and contributing to cell lysis. SEM analysis has shown significant morphological alterations, including shape distortion and surface roughness, in *S. aureus* cells treated with ZnO-NPs [78,91]. Additionally, ZnO-NPs can disrupt established biofilms by downregulating biofilm-related genes, such as *icaA* and *fnbA*, which are essential for biofilm development [86,92]. The combined effects of ROS generation and biofilm matrix disruption further destabilize biofilms, increasing bacterial susceptibility to antimicrobial treatments. Therefore, ZnO-NPs exhibit multifaceted antibacterial and antibiofilm activities against *S. aureus*, involving the release of Zn^2+^ ions, ROS generation, electrostatic interactions, and biofilm inhibition. These mechanisms make ZnO-NPs promising alternatives or adjuncts to traditional antibiotics, particularly in addressing the growing challenge of antibiotic resistance. Further studies into the specific molecular pathways affected by ZnO-NPs will be crucial for optimizing their applications in food safety.

## 4. Conclusions

The rise of multidrug-resistant (MDR) pathogens, particularly *S. aureus*, in marketed fish, presents a critical challenge to food safety and public health. This study introduces an innovative eco-friendly method for biosynthesizing ZnO-NPs using *S. rebaudiana* extracts, showing their impressive antibacterial and antibiofilm activities against MDR *S. aureus* strains isolated from fish and their mechanism. By employing *S. rebaudiana* as a reducing and capping agent, this study presents a sustainable alternative to traditional chemical synthesis. The obtained data reveal that these ZnO-NPs interrupt *S. aureus* bacterial cell membranes and induce DNA fragmentation, effectively leading to bacterial death and significantly inhibiting biofilm formation. With inhibition zones reaching 24.4 mm and biofilm inhibition rates of up to 92.1%, the green-synthesized ZnO-NPs emerge as an effective agent for reducing the contamination of MDR strains in marketed fish. This study contributes valuable mechanistic insights and opens avenues for sustainable antimicrobial strategies to tackle the escalating threat of antibiotic resistance. Future research should evaluate nanoparticle safety and efficacy in vivo and explore their broader applications in food safety and clinical environments.

## Figures and Tables

**Table 1:** ZnO-NPs antibacterial potential against the selected pathogenic MDR *S. aureus* strains.

Isolate Code	Biofilm Inhibition (%)					
	0 µg	50 µg	100 µg	150 µg	200 µg	250 µg
MFSE-M-1	0.0 ± 0.0 ^a^*	21.2 ± 1.3 ^a^	40.3 ± 2.1 ^abc^	53.5 ± 1.3 ^abc^	80.3 ± 1.2 ^ac^	89.4 ± 1.1 ^a^
MFSE-NT-21	0.0 ± 0.0 ^a^	27.1 ± 0.9 ^b^	37.7 ± 1.5 ^b^	50.3 ± 1.6 ^b^	74.4 ± 1.5 ^bd^	82.1 ± 2.5 ^b^
MFSE-S-49	0.0 ± 0.0 ^a^	31.8 ± 1.4 ^c^	44.3 ± 2.2 ^c^	56.6 ± 1.8 ^c^	77.3 ± 2.4 ^ab^	85.5 ± 2.3 ^abc^
MFSE-S-11	0.0 ± 0.0 ^a^	16.3 ± 1.5 ^d^	22.3 ± 2.5 ^d^	42.5 ± 2.4 ^d^	64.7 ± 1.5 ^e^	72.2 ± 1.2 ^d^
MFSE-S-4	0.0 ± 0.0 ^a^	45.1 ± 0.9 ^e^	54.3 ± 3.2 ^e^	63.8 ± 1.0 ^e^	80.9 ± 1.2 ^ac^	90.7 ± 2.2 ^a^
MFSE-S-20	0.0 ± 0.0 ^a^	19.3 ± 2.3 ^ad^	27.5 ± 2.3 ^f^	46.9 ± 1.9 ^b^	70.9 ± 0.6 ^df^	78.8 ± 1.1 ^b^
MFSE-M-8	0.0 ± 0.0 ^a^	36.5 ± 2.2 ^f^	46.7 ± 1.5 ^c^	56.4 ± 1.4 ^c^	78.9 ± 1.0 ^a^	88.9 ± 2.0 ^a^
MFSE-M-3	0.0 ± 0.0 ^a^	40.7 ± 2.5 ^f^	55.2 ± 3.0 ^e^	64.7 ± 1.6 ^e^	84.6 ± 1.4 ^c^	92.1 ± 1.7 ^a^
MFSE-NT-35	0.0 ± 0.0 ^a^	27.3 ± 1.2 ^b^	35.7 ± 2.5 ^b^	49.9 ± 1.3 ^b^	73.2 ± 1.9 ^bf^	81.5 ± 1.4 ^bc^
*p*-value	NA	0.975	0.957	0.974	0.923	0.925

* Different letters indicate statistically significant differences between isolates and treatments.

**Table 2:** Antibiofilm potential of ZnO-NPs towards resistance and virulence gene-producing *S. aureus strains* isolated from marketed fish species.

Isolate Code	Biofilm Inhibition (%)					
	0 µg	50 µg	100 µg	150 µg	200 µg	250 µg
MFSE-M-1	0.0 ± 0.0 ^a^*	21.2 ± 1.3 ^a^	40.3 ± 2.1 ^abc^	53.5 ± 1.3 ^abc^	80.3 ± 1.2 ^ac^	89.4 ± 1.1 ^a^
MFSE-NT-21	0.0 ± 0.0 ^a^	27.1 ± 0.9 ^b^	37.7 ± 1.5 ^b^	50.3 ± 1.6 ^b^	74.4 ± 1.5 ^bd^	82.1 ± 2.5 ^b^
MFSE-S-49	0.0 ± 0.0 ^a^	31.8 ± 1.4 ^c^	44.3 ± 2.2 ^c^	56.6 ± 1.8 ^c^	77.3 ± 2.4 ^ab^	85.5 ± 2.3 ^abc^
MFSE-S-11	0.0 ± 0.0 ^a^	16.3 ± 1.5 ^d^	22.3 ± 2.5 ^d^	42.5 ± 2.4 ^d^	64.7 ± 1.5 ^e^	72.2 ± 1.2 ^d^
MFSE-S-4	0.0 ± 0.0 ^a^	45.1 ± 0.9 ^e^	54.3 ± 3.2 ^e^	63.8 ± 1.0 ^e^	80.9 ± 1.2 ^ac^	90.7 ± 2.2 ^a^
MFSE-S-20	0.0 ± 0.0 ^a^	19.3 ± 2.3 ^ad^	27.5 ± 2.3 ^f^	46.9 ± 1.9 ^b^	70.9 ± 0.6 ^df^	78.8 ± 1.1 ^b^
MFSE-M-8	0.0 ± 0.0 ^a^	36.5 ± 2.2 ^f^	46.7 ± 1.5 ^c^	56.4 ± 1.4 ^c^	78.9 ± 1.0 ^a^	88.9 ± 2.0 ^a^
MFSE-M-3	0.0 ± 0.0 ^a^	40.7 ± 2.5 ^f^	55.2 ± 3.0 ^e^	64.7 ± 1.6 ^e^	84.6 ± 1.4 ^c^	92.1 ± 1.7 ^a^
MFSE-NT-35	0.0 ± 0.0 ^a^	27.3 ± 1.2 ^b^	35.7 ± 2.5 ^b^	49.9 ± 1.3 ^b^	73.2 ± 1.9 ^bf^	81.5 ± 1.4 ^bc^
*p*-value	NA	0.975	0.957	0.974	0.923	0.925

* Means with the same letters in the same column showed an insignificant difference.

## Data Availability

The data presented in this study are available upon request from the corresponding author.

## Supplementary Materials

The following supporting information can be downloaded at: https://www.mdpi.com/article/10.3390/nano15050369/s1, Figure S1. Neighbor-joining tree constructed based on the phylogeny analysis of the selected MDR *S. aureus* strains that produce resistance and virulence genes. The tree highlights their position among closely related taxa. The numbers on the branches represent bootstrap values (>50%) based on 500 replicates, indicating the confidence level of the phylogenetic groupings.; Figure S2: The statistical analysis using *t*-test of antibiofilm activity of ZnO-NPs against pathogenic MDR *S. aureus* strains isolated from retail fish.; Table S1. Antimicrobial agents and zone interpretive chart based on CLSI guidelines (CLSI, 2017); Table S2. Primers and PCR amplification conditions used in this study; Table S3. Primers used for the molecular identification and characterization of MDR *S. aureus* strains.; Table S4. The susceptibility of *S. aureus* strains in tested marketed fish to different antibacterial agents.; Table S5. The drug resistance patterns (DRPs) of MDR *S. aureus* isolated from different marketed fish.
