# Supplementary material for: Harnessing Stevia rebaudiana for Zinc Oxide Nanoparticle Green Synthesis: A Sustainable Solution to Combat Multidrug-Resistant Bacterial Pathogens
Source: Nanomaterials (Basel). 2025 Feb 27;15(5):369. doi: 10.3390/nano15050369 (PMC11901501; doi:10.3390/nano15050369)
Supplement: Supplementary file 1 [file nanomaterials-15-00369-s001.zip › nanomaterials-3444281-supplementary.pdf]

Supplementary data

Harnessing *Stevia rebaudiana* for Zinc Oxide Nanoparticles Green Synthesis: A Sustainable Solution to Combat Multidrug-Resistant Bacterial Pathogens

Mohamed Tharwat Elabbasy[1*], Rasha M. El Bayomi[2*], Esraa A. Abdelkarim[2*], Abd El-Salam E. Hafez[2], Mohamed S. Othman[3], Mohamed E. Ghoniem[4], Mai A. Samak[1], Muteb H. Alshammari[5], Fahad Awwadh Almarshadi[6], Tamer Elsamahy[7*], Mohamed A. Hussein[2*]

[1]Department of Pathology, College of Medicine, University of Ha'il, Ha'il 55476, Saudi Arabia
[2]Food Hygiene, Safety, and Technology Department, Faculty of Veterinary Medicine, Zagazig University, Zagazig 44511, Egypt
[3]Department of Biochemistry, College of Medicine, University of Ha'il, Ha'il 55476, Saudi Arabia
[4]Department of Internal Medicine, College of Medicine, University of Ha'il, Ha'il 2240, Saudi Arabia
[5]Department of Health Informatics, College of Public Health and Health Informatics, University of Ha'il, Ha'il 55476, Saudi Arabia
[6]Department of Public Health, College of Public Health and Health Informatics, University of Ha'il, Ha'il 55476, Saudi Arabia
[7]Independent Researcher, Zhenjiang 212013, China
.
* Correspondence: Esraaadel92t@gmail.com (E.A.A.); tamer63916@science.tanta.edu.eg (T.E.)

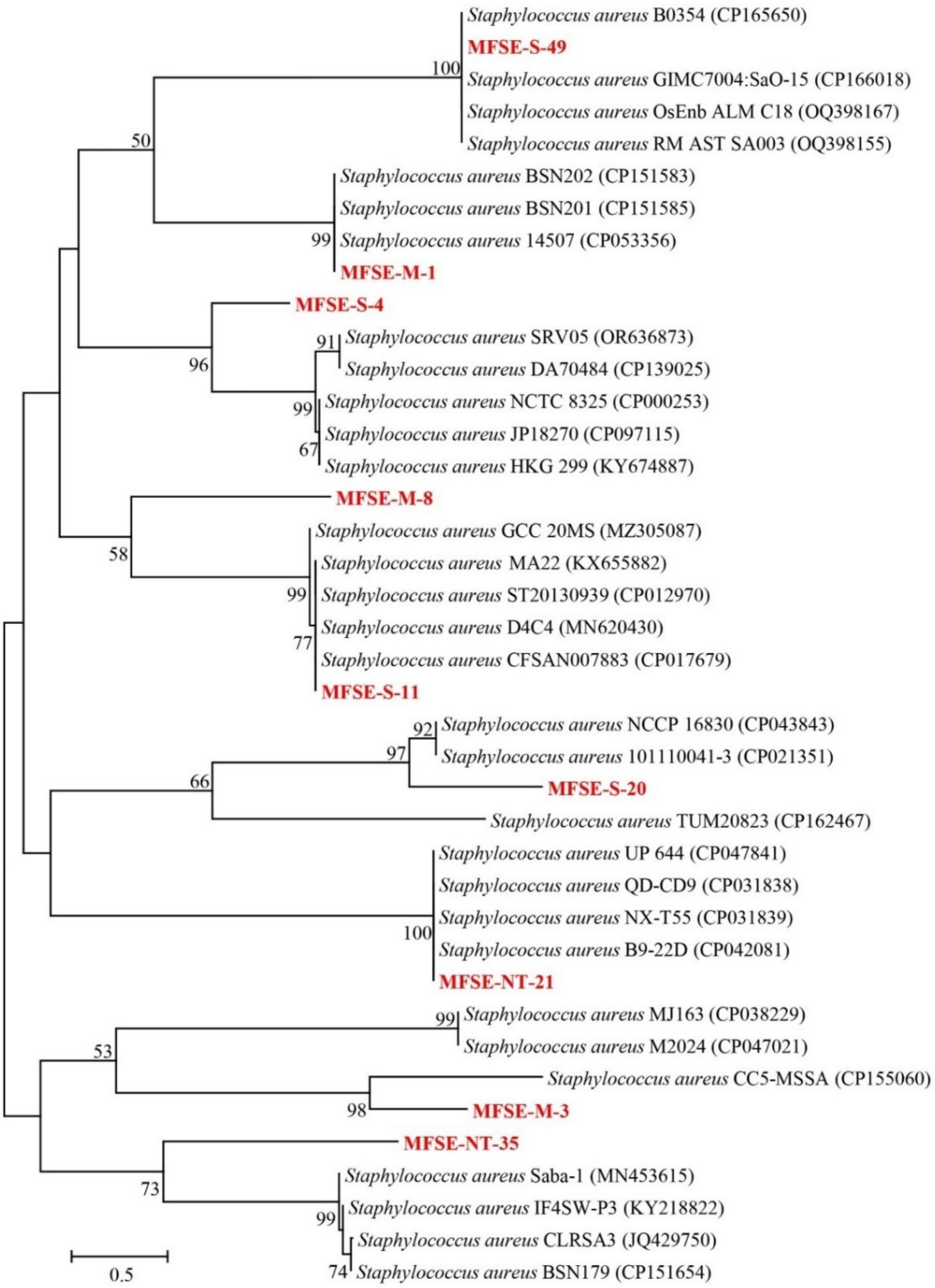

Figure S1. Neighbour-Joining tree constructed based on the phylogeny analysis of the selected MDR *S. aureus* strains that produce resistance and virulence genes. The tree highlights their position among closely related taxa. The numbers on the branches represent bootstrap values (>50%) based on 500 replicates, indicating the confidence level of the phylogenetic groupings.

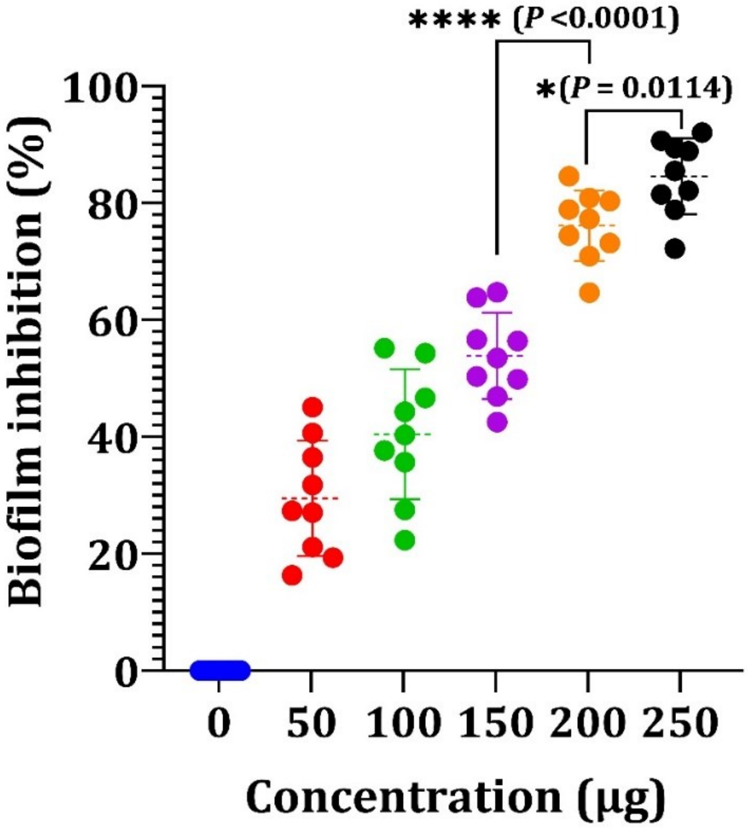

Figure S2. The statistical analysis using t-test of antibiofilm activity of ZnO-NPs against pathogenic MDR *S. aureus* strains isolated from retail fish.

Table S1. Antimicrobial agents and zone interpretive chart based on CLSI guidelines (CLSI, 2017).

Antimicrobial group	Antimicrobial agent	Abbreviations	Concentration range (μg/ml)	Breakpoints		
				S	I	R
Aminoglycosides	Amikacin	AMK	30	≥17	15-16	≤14
	Gentamicin	GEN	10	≥15	13-14	≤12
Cephalosporins	Cefalexin	CFX	30	≥21	19-20	≤18
	Cefpodoxime	CFP	10	≥21	18-20	≤17
Chloramphenicol	Chloramphenicol	CHL	30	≥18	13-17	≤12
Fluroquinolone	Enrofloxacin	ENR	5	≥21	17-20	≤16
	Marbofloxacin	MAR	5	≥20	15-19	≤14
Carbapenem	Imipenem	IMI	10	≥23	20-22	≤19
Polymixins	Polymyxin B	POL	300	≥12	9-11	≤8
Tetracycline	Tetracycline	TET	30	≥15	12-14	≤11
Sulfonamide	Trimethoprim-Sulfamethoxazole	SXT	1.25/23.75	≥30	26-29	≤25
β-lactam	Piperacillin	PIP	100	≥21	18-20	≤17

Table S2. Primers and PCR amplification conditions were used in this study.

Primers	Orientation	Sequence (5'-3')	PCR amplification conditions
**27F**	Forward	AGAGTTTGATCYTG GCTCAG	*Initial denaturation: 95°C for 5min
**1492R**	Reverse	TACGGTTACCTTGTTACGACTT	*Denaturation: 35 cycles of heating at 94°C for 30 sec
			*Annealing: 54°C for 30 sec
			*Extension: 72°C for 3 min
			*Final extension: 72°C for 10 min.

Table S3. Primers used for the molecular identification and characterization of MDR *S. aureus* strains.

Target gene	Orientation	Sequence (5′-3′)	References
sea	Forward	GGTTATCAATGTGCGGGTGG	Mehrotra et al.
	Reverse	CGGCACTTTTTTCTCTTCGG	(2000)
seb	Forward	GTATGGTGGTGTAACTGAGC	
	Reverse	CCAAATAGTGACGAGTTAGG	
sec	Forward	AGATGAAGTAGTTGATGTGTATGG	
	Reverse	CACACTTTTAGAATCAACCG	
sed	Forward	CCAATAATAGGAGAAAATAAAAG	
	Reverse	ATTGGTATTTTTTTTCGTTC	
see	Forward	AGGTTTTTTCACAGGTCATCC	
	Reverse	CTTTTTTTTCTTCGGTCAATC	
*fnb*A	Forward	GTGAAGTTTTAGAAGGTGGAAAGATTAG	Tristan et al.
	Reverse	GCTCTTGTAAGACCATTTTTCTTCAC	(2003)
*fnb*B	Forward	GTAACAGCTAATGGTCGAATTGATACT	
	Reverse	CAAGTTCGATAGGAGTACTATGTTC	
clfa	Forward	ATTGGCGTGGCTTCAGTGCT	
	Reverse	CGTTTCTTCCGTAGTTGCATTTG	
tst	Forward	TTCACTATTTGTAAAAGTGTCAGACCCACT	Li et al. (2018)
	Reverse	TACTAATGAATTTTTTTATCGTAAGCCCTT	
pvl	Forward	ATCATTAGGTAAAATGTCTGGACATGATCCA	
	Reverse	GCATCAASTGTATTGGATAGCAAAAGC	
*aad*E	Forward	AGTTAGATAATTACCTAAAGGGCG	Argudín et al.
	Reverse	ATATCAGCGGCATATGTGCTATCC	(2012)
*aac*A-*aph*D	Forward	TAATCC AAG AGC AAT AAG GGC	Strommenger et
	Reverse	GCCACACTATCATAACCACTA	al. (2003)
*mec*A	Forward	GCGATTGATGGTGATACGGTT	Azimian et al.
	Reverse	AGCCAAGCCTTGACGAACTAAAGC	(2012)
cfr	Forward	GTGAAGCTCTAGCCAACCGTC	Osman et al.
	Reverse	GCAGCGTCAATATCAATCCC	(2019)
*bla*Z	Forward	ACTTCAACACCTGCTGCTTTC	Martineau et al.
	Reverse	TGACCACTTTTATCAGCAACC	(2002)
spc	Forward	ACCAAATCAAGCGATTAAA	Fessler et al.
	Reverse	GTCACTGTTTGCCACATTCG	(2010)
*apm*A	Forward	CGTTTGCTTCGTGCATTAAA	Fessler et al.
	Reverse	TTGACACGAAGGAGGGTTTC	(2011)

Table S4. The susceptibility of *S. aureus* strains in tested marketed fish to different antibacterial agents.

Antibacterial agent	Resistant		Intermediate		Susceptible	
	No.	(%)	No.	(%)	No.	(%)
Amikacin (AMK)	116	67.8	6	3.51	49	28.7
Gentamicin (GEN)	124	72.5	5	2.92	42	24.6
Cefalexin (CFX)	53	31.0	5	2.92	113	66.1
Cefpodoxime (CEP)	76	44.4	1	0.58	94	55.0
Chloramphenicol (CHL)	56	32.7	4	2.34	111	64.9
Marbofloxacin (MAR)	97	56.7	4	2.34	70	40.9
Enrofloxacin (ENR)	63	36.8	3	1.75	105	61.4
Imipenem (IMI)	73	42.7	1	0.58	97	56.7
Polymyxin B (POL)	88	51.5	5	2.92	78	45.6
Tetracycline (TET)	89	52.0	2	1.17	80	46.8
Piperacillin (PIP)	76	44.4	4	2.34	91	53.2
Trimethoprim-sulfamethoxazole (SXT)	71	41.5	6	3.51	94	55.0

Table S5. The drug resistance patterns (DRPs) of MDR *S. aureus* isolated from different marketed fish.

Pattern code	DRP	Isolate number	MAR index	Pattern code	DRP	Isolate number	MAR index
P1	GEN, CEP, CHL	3	0.25	P5b	Y, AMK, GEN, MAR, TET	4	0.58
P1a	GEN, MAR, ENR	3	0.25	P5c	Y, GEN, CFX, CEP, MAR	7	0.58
P2	AMK, GEN, IMI, SXT	4	0.33	P6	W, CEP, CHL, POL, TET, SXT	7	0.67
P2a	AMK, MAR, POL, TET	4	0.33	P6a	Y, AMK, GEN, MAR, TET, PIP	6	0.67
P3	AMK, CFX, MAR, PIP, SXT	5	0.42	P6b	GEN, CFX, CEP, CHL, IMI, POL, TET, PIP	3	0.67
P3a	AMK, GEN, CEP, CHL, IMI	5	0.42	P7	W, Y, CEP, MAR, TET	3	0.75
P3b	AMK, GEN, IMI, POL, TET	5	0.42	P7a	X, AMK, GEN, ENR, POL, PIP, SXT	6	0.75
P4	Z, AMK, ENR, IMI,	6	0.50	P8	W, Z, CEP, MAR, IMI, POL,	8	0.83
P4a	W, CEP, CHL, PIP	6	0.50	P8a	W, Z, CEP, MAR, IMI, POL	4	0.83
P4b	Z, GEN, MAR, POL,	6	0.50	P9	X, Y, Z, AMK, GEN	3	0.92
P5	X, AMK, GEN, ENR, PIP	7	0.58	P9a	X, Y, Z, AMK, CFX	2	0.92
P5a	Z, AMK, GEN, CEP, POL	7	0.58	P10	W, X, Y, Z	8	1.00

W, AMK, GEN, CFX; **X**, CEP, CHL, MAR; **Y,** ENR, IMI, POL; **Z**, TET, PIP, SXT.

References

Argudín, M.A., Mendoza, M.C., Gonz´alez-Hevia, M.A., Bances, M., Guerra, B., Rodicio, M.R., 2012. Genotypes, exotoxin gene content, and antimicrobial resistance of *Staphylococcus aureus* strains recovered from foods and food handlers. Appl. Environ. Microbiol. 78, 2930–2935.

Azimian, A., Havaei, S.A., Fazeli, H., Naderi, M., Ghazvini, K., Samiee, S.M., 2012. Genetic characterization of a vancomycin-resistant *Staphylococcus aureus* isolate from the respiratory tract of a patient in a university hospital in northeastern Iran. J. Clin. Microbiol. 50 (11), 3581–3585.

CLSI, 2017. Performance standards for antimicrobial susceptibility testing. In: CLSI Supplement M100, 27th edn. Clinical Laboratory Standards Institute, Wayne, PA.

Fessler, A., Kadlec, K., Schwarz, S., 2011. Novel apramycin resistance gene apmA in bovine and porcine methicillin-resistant Staphylococcus aureus ST398 isolates. J. Antimicrob. Chemother. 55, 373–375.

Fessler, A., Scott, C., Kadlec, K., Ehricht, R., Monecke, S., Schwarz, S., 2010. Characterization of methicillin-resistant Staphylococcus aureus ST398 from cases of bovine mastitis. J. Antimicrob. Chemother. 65, 619–625.

Li, X., Fang, F., Zhao, J., Lou, N., Li, C., Huang, T., Li, Y., 2018. Molecular characteristics and virulence gene profiles of *Staphylococcus aureus* causing bloodstream infection. Braz. J. Infect. Dis. 22 (6), 487–494.

Martineau, F., Picard, J.F., Lansac, N., Menard, C., Roy, H.P., Ouellette, M., Bergeron, G. M., 2002. Correlation between the resistance genotype determined by multiplex PCR assays and the antibiotic susceptibility patterns of Staphylococcus aureus and Staphylococcus epidermidis. Antimicrob. Agents Chemother. 44 (2), 231–238.

Mehrotra, M., Wang, G., Johnson, W.M., 2000. Multiplex PCR for detection of genes for *Staphylococcus aureus* enterotoxins, exfoliative toxins, toxic shock syndrome toxin 1, and methicillin resistance. J. Clin. Microbiol. 38, 1032–1035.

Osman, K.M., Badr, J., Orabi, A., Elbehiry, A., Aalaa Saad, A., Ibrahim, M.D.S., Hanafy, M.H., 2019. Poultry as a vector for emerging multidrug resistant *Enterococcus* spp.: first report of vancomycin (van) and the chloramphenicol–florfenicol (cat-fex-cfr) resistance genes from pigeon and duck faeces. Microb. Pathog. 128, 195–205.

Strommenger, B., Kettlitz, C., Werner, G., Witte, W., 2003. Multiplex PCR assay for simultaneous detection of nine clinically relevant antibiotic resistance genes in *Staphylococcus aureus*. J. Clin. Microbiol. 41, 4089–4094.

Tristan, A., Ying, L., Bes, M., Etienne, J., Vandenesch, F., Lina, G., 2003. Use of multiplex PCR to identify *Staphylococcus aureus* adhesins involved in human hematogenous infections. J. Clin. Microbiol. 41, 4465–4467.
